# VNS in drug resistant epilepsy: preliminary report on a small group of patients

**DOI:** 10.1186/1824-7288-36-30

**Published:** 2010-04-14

**Authors:** Emilio Franzoni, Valentina Gentile, Maria Chiara Colonnelli, Daniela Brunetto, Ilaria Cecconi, Luisa Iero, Filomena C Moscano, Duccio M Cordelli, Valentina Marchiani

**Affiliations:** 1 Child Neuropsychiatry Unit, Bologna University, Italy; 2 PhD School Applied Neurological Sciences, Siena University, Italy

## Abstract

**Background:**

In 1997 Vagus Nerve Stimulation (VNS) received approval from the US Food and Drug Administration (FDA) as an adjunctive therapy in the treatment of medically intractable partial epilepsy in people aged 12 years and older who are ineligible for resective epilepsy surgery. Although the exact mechanisms of action are unknown, the use of VNS with children has increased, including those younger than 12 years of age, or those with generalized epilepsy.

**Methods:**

We describe the outcome for the first group of nine patients, aged 8-28 years, who had pharmaco-resistant epilepsy and were treated with VNS. During the follow up, we gradually and slowly increased the parameters of the stimulation in order to assess the efficacy of VNS even at parameters which would usually be considered "non-therapeutic", along with possible side effects and changes in quality of life.

**Results:**

At the last follow, up 1 patient was "seizures free", 3 were "very good responders", 3 were "good responders" and 2 were "non responders". We obtained an initial seizure reduction with low stimulation parameters, the highest current reached being 2.00 mA. This observation supports the possibility that, for younger patients, lower stimulation intensities than those commonly used in clinical practice for adults can be therapeutic. We also wanted to underline the reduction in seizure frequency (~91,7%) and the reduction in seizure duration (> 50%) in the patients affected by drug-resistant absence epilepsy. Adverse effects were mild, tolerable and, in most of cases, easily resolved by adjusting the stimulation parameters. Hoarseness of voice was the most frequent side effect. The improvements in the quality of life are relevant and seem to be independent of the VNS effect in controlling seizures.

**Conclusions:**

Our small experience seems to confirm the efficacy and safety of VNS in drug resistant partial and generalized epilepsy in developing age groups.

## Background

In 1997 Vagus Nerve Stimulation (VNS) received approval from the US Food and Drug Administration (FDA) as an adjunctive therapy in the treatment of medically intractable partial epilepsy in people aged 12 years and older [1,2] who are ineligible for resective epilepsy surgery [3,4]. Although the exact mechanisms of action are unknown, the use of VNS in children has increased, including in those aged under 12 and in those with generalized epilepsy. It is evident that VNS offers substantial therapeutic benefits to some patients, without causing major side effects [3]. VNS is not usually associated with the common central nervous system side effects, such as dizziness, ataxia, insomnia, cognitive impairment, or weight gain [5], which sometimes limit the use of AEDs.

We describe the outcome for the first group of patients with pharmaco-resistant epilepsy who were treated with VNS at the Child Neuropsychiatric Unit of S.Orsola-Malpighi Hospital, University of Bologna (Italy).

## Materials and methods

Our study included nine patients, five females and four males, aged 8-28 years with pharmaco-resistant epilepsy (Table 1). Patient 1 presents absence epilepsy, patient 2 and 3 present partial epilepsy secondary to herpetic encephalitis with drop attacks, patient 4 presents partial epilepsy due to a double cortex, patient 5 and 7 present partial epilepsy probably symptomatic, patient 6 and 8 present symptomatic partial epilepsy due to peri-natal sufferance, patient 9 is affected by Lennox-Gastaut syndrome.

**Table 1:** Our patients

Etiology	Age	Duration of epilepsy	Seizure Type	VNS stimulation parameters associated with seizures reduction	Syndrome	Follow-up	Actual VNS stimulation parameters	Seizures frequency
1 Idiopathic	16 y	9 y	Generalised seizures	I: 1.75 mA; T off: 5 min	Absence epilepsy	29 m	I: 1.75 mA; T off: 5 min	Very good responder > 75%

2 Symptomatic	8 y	7 y	Drop attacks, partial seizures with secondary generalization	i: 2.00 mA; T off: 5 min	Partial epilepsy secondary to herpetic encephalitis	29 m	i: 2.00 mA; T off: 5 min	Good responder > 50%

3 Symptomatic	11 y	10 y	Drop attacks, partial seizures	i: 1.25 mA; T off: 5 min	Partial epilepsy secondary to herpetic encephalitis	26 m	i: 1.50 mA; T off: 5 min	Very good responder >75%

4 Symptomatic	26 y	17 y	Partial	?	Partial epilepsy due to double cortex	22 m	i: 1.50 mA; F: 20 Hz T off: 5 min	Non responder

5 Probably Symptomatic	11 y	11 y	Partial	i: 1.50 mA; T off: 40 min	Probably symptomatic epilepsy	22 m	i: 1.50 mA; T off: 5 min	Good responder > 50%

6 Symptomatic	28 y	28 y	Partial seizures with secondary generalization	I: 1.25 mA; T off: 180 min	Symptomatic partial epilepsy due to peri-natal sufferance	13 m	i: 2.00 mA; T off: 25 min	Non responder

7 Probably Symptomatic	23 y	4 y	Partial seizures with secondary generalization	i: 0.50 mA; T off: 180 min	Probably symptomatic epilepsy	10 m	i: 1.00 mA; T off: 45 min	Seizures free

8 Symptomatic	17 y	11 y	Partial seizures with secondary generalization	i: 1.25 mA; T off: 120 min	Symptomatic partial epilepsy due to peri-natal sufferance	8 m	i: 1.25 mA; T off: 90 min	Very good responder >75%

9 Probably Symptomatic	14 y	14 y	Partial seizures with secondary generalization	i: 1.25 mA; T off: 120 min	Lennox-Gastaut syndrome	6 m	i: 1.25 mA; T off: 90 min	Very good responder >75%

**Initial VNS stimulation parameters: **i: 0.25 mA, F: 30 Hz, A: 500 μs, T on: 30 s, T off: 180 min

m: months

y: years

They had received from 7 to 15 different anti-epileptic drugs and were not eligible for surgical resection. VNS was implanted in the period June 2007- June 2009. The follow-up lasted 5 to 29 months. After VNS implantation, patients had clinical examinations every 15-30 days in which we evaluated the frequency, intensity and duration of their seizures, the side effects, and any changes in their quality of life, on the basis of a diary filled in by the family, without the support of any standardized questionnaires. During the follow up, we regulated the stimulation parameters, which were gradually and slowly increased, in order to assess the efficacy of VNS at parameters which would usually be considered "non-therapeutic" [3]. The efficacy of the treatment was measured as the percentage change in seizures at each visit. The stimulation parameters were adjusted in line with standard medical practice for VNS implanted patients [5]. Initial stimulation parameters were: output current = 0.25 mA, frequency = 30 Hz, pulse width = 500 μs, signal on-time = 30 sec and off-time = 180 minutes. The stimulus intensity was increased stepwise by 0.25 mA up to a maximum of 2.00. In addition, the off-time was reduced from 180 minutes to 5 minutes. We did not change the frequency or the duration of the stimulus, other than in two cases in which there was the onset of side effects (hoarseness). As regards Quality of Life (QOL), we considered improvements in alertness, verbal communication, memory, school/professional achievement, mood, and reduction in post-ictal state and in seizure clustering [5]. No changes to drugs were made, other than for two patients who experienced a worsening in their clinical patterns.

## Results

No initial surgical complications were reported in this cohort of patients and no complications caused by implantation were reported. All children were known to have the devices in place and functioning.

Only 3 months from implantation, drop attacks had disappeared in one patient and decreased in another. From the third month of treatment, patient 1 (table 1) with drug-resistant absence epilepsy experienced a reduction in the intensity and duration of absences, and this positive result remained constant. After nine months of treatment, the patient showed a 91,7% reduction in seizure frequency and a reduction in seizure duration of more than 50% (from 20-40 s down to 6-20 s). These data were confirmed at follow-up over 29 months.

At the last follow up, 1 patient was "seizure free" with a follow-up duration of 10 months, 4 were "very good responders" because of a reduction in fits of more than 75%, 2 of these with a follow-up duration more than 12 months. 2 patients were "good responders" with a seizure reduction of more than 50%, both with a follow-up duration more than 12 months; 2 patients were "non-responders" after a follow-up duration more than 12 months.

Magnet activation was used by the patients or by caregivers in case of frequent seizures. One patient reported that the magnet was effective in aborting or decreasing the intensity or duration of her seizures. Two patients reported that the magnet had no effect, while two patients had not yet used the magnet.

The improvement in the quality of life was particularly relevant for 7 patients, and this seemed to be independent of the VNS effect in controlling seizures. No specific test was used.

**Figure 1:**
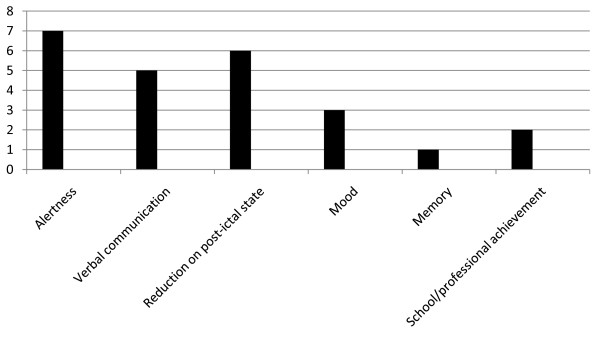
**Improvement in Quality of Life (QOL) in our patients**.

In general, the best results were achieved in alertness (7 patients), verbal communication (5 patients) and in reduction in post-ictal state (6 patients) (Figure 1).

From the third month of treatment, 2/9 patients showed an increase in alertness, verbal communication and reduction in post-ictal state, 2/9 in alertness and reduction in post-ictal state, 1/9 in alertness, verbal communication, mood and reduction in post-ictal state, 1/9 in alertness, verbal communication, mood, reduction in post-ictal state and school achievement, and 1/9 in alertness, verbal communication, memory, mood and school achievement. In contrast, 2/9 showed no increase in QOL, one of whom continued to complain of severe weariness, this probably being caused by a recently diagnosed and as yet untreated hypothyroidism.

In our patients these improvements in QOL were reported by parents even if they did not referred a reduction of seizures. This suggests that the improvements in QOL may be independent of the anti-seizure effect that VNS has.

## Discussion

The longest follow-up in this series was 29 months; therefore, the results are still preliminary.

Our small experience with VNS seems to confirm the efficacy of the treatment in the reduction of the frequency and intensity of seizures in drug-resistant epilepsy at developing age. Multiple studies have shown a slightly higher response rate in children as compared with adults, and the longer-term follow-ups (> 5 years) in children have shown a 50% decrease in seizure frequency in about 60% of patients, with a 75% decrease in 40% of patients, though overall numbers are still low [6].

In accordance with other experiences [7,8], we observed an initial decrease in daily seizure frequency and the occurrence of seizure-free days. As time went by, more seizure-free days and fewer seizure days were achieved, with decreased severity and reduction in post-ictal state. Sometimes seizure-free periods coalesced into weeks, with seizure days becoming more and more distant from each other. On the same grounds there seemed to be a reasonable reduction in the utilization of healthcare services, with less accesses in the emergency room, and the time spent on epilepsy-related tasks [6].

The low number of patients did not allow us to evaluate the type of seizure which is most responsive to VNS therapy, though it should be noted that after only 3 months of therapy, drop attacks stopped in one patient and were reduced in one, this observation confirming the notable efficacy of VNS therapy in controlling drop attacks [3,4,7].

**Figure 2:**
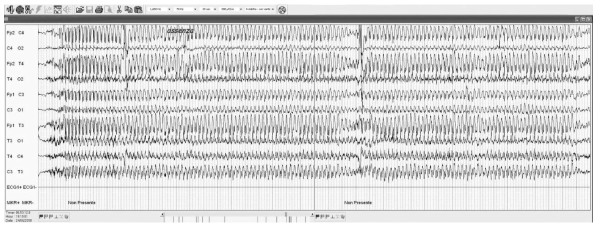
**EEG of a patient before VNS implantation**.

**Figure 3:**
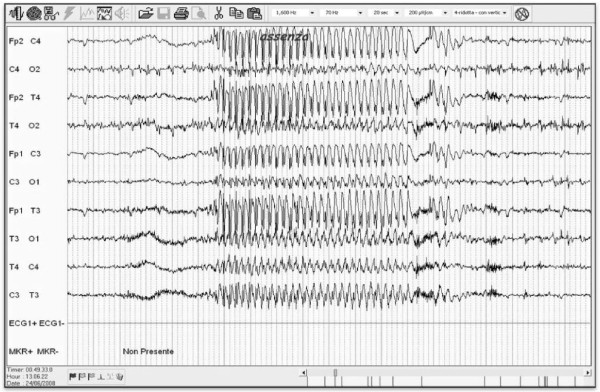
**EEG of a patient after VNS implantation**.

We would underline the fact that patient 1 (table 1) presenting with absence drug-resistant epilepsy showed a reduction in seizure frequency of 91,7% and a reduction in seizure duration of more than 50% after 9 months of treatment. Before the implantation seizures duration was from 20 to 40 s in every EEGs, whereas after VNS implantation it was lower, however never longer than 20 s (Figure 2, 3). This datum was confirmed after 29 months of follow-up. Until now, there have been few reports showing the effects of VNS in patients with absences [9-11], which are not considered the main indication for VNS treatment. This patient satisfied diagnostic criteria for Childhood Absence Epilepsy (CAE), characterized by the occurrence of frequent absences in otherwise normal children. The age of onset is around 6-8 years and the ictal EEG classically shows paroxysms of generalized bilateral synchronous 3 Hz spike-waves on a normal background activity [12]. Exact descriptions of the course and prognosis of childhood epilepsies, including CAE, have been inconsistent in earlier studies, probably because of methodological differences and variations in patient inclusion criteria [12]. Most studies classified the absence epilepsy according to the criteria of the ILAE of 1989 [13] or the criteria as defined by Loiseau in 1992 [14]. It was observed that a patient fulfilling the stricter criteria of ILAE had significantly higher remission rates, fewer GTCS, and shorter mean treatment periods. These strict criteria have been accepted by the ILAE Task Force for Classification and Terminology in 2005 [15] and in a study of 2007 was confirmed that children fulfilling them, have a significantly better outcome [16].

Earlier age at onset, the appearance of generalized tonic-clonic seizures (GTCSs), difficult and incomplete response to treatment, and the absence of seizure with focal onset of abnormalities, were considered to be factors indicating an unfavorable prognosis [12,17,18]. The pharmaco-resistance was confirmed in this patient by the fact that various drugs (more than 5) were used. EEG activity and the clinical features of our patient suggested an unfavorable prognosis. We observed long discharges at 3 Hz with frontal onset (Figure 2). These data associated with a long duration of epilepsy suggest that a natural improvement in the course of epilepsy is not frequent, in particular in this kind of patient.

Another goal of our observation was to attempt to evaluate the efficacy of VNS with lower stimulation parameters.

The level of the stimulation parameters is still controversial and there are no guide-lines, only some indications, these being not to exceed a stimulation current of 2,00 mA and to keep a standard cycle of 5 min OFF and 3 min ON. We obtained an initial reduction in seizures using low stimulation parameters (stimulation current of 1,25 mA; ON 30 sec, OFF 120 min) in patients n°8 and n°9, who were very good responders (Table 1).

The patient who was seizures-free obtained an initial reduction in seizures with the following parameters: stimulation current of 0,50 mA; ON 30 sec, OFF 180 min. At the time of writing, the stimulation current being used was 1.00 mA.

Patient n°4 was a non responder: we did not report the initial VNS stimulation parameters because we did not see any improvement in the frequency of seizures, although we reached a higher stimulation current (Table 1).

This observation suggests the possibility that lower stimulation intensities can be therapeutic, not only for children but also in young patients.

Adverse effects were mild, tolerable and, in most of cases, easily resolved by adjusting stimulation parameters in our patients. These side effects appear particularly mild and infrequent, above all, if compared with the undesirable effects caused by the anti-epileptic drugs taken by the patients. The most frequent side effect was hoarseness of voice during the ON periods of stimulation. This discomfort was persistent in one patient, whereas in four others hoarseness of voice was reduced by modifying the pulse width. In another patient, we noticed a partial escape from the pocket of bipolar lead in the chest, though device functioning was not compromised. The child developed a hypertrophic chest scar. In three cases, no VNS-related side effects were reported.

VNS side effects can also include weight loss [19]. The regulation of feeding behavior is complex and poorly understood, and the effect of VNS therapy on body weight is unclear: VNS can cause weight loss by engaging vagal afferents from the gastrointestinal tract which mediates satiety [19]. Animal experiments have shown that stimulation of the vagus nerve effectively reduces eating, with a corresponding weight loss [20]. These type of studies have not been performed in humans and it may be necessary to design a vagal pacing therapy that mimics vagal activity in fasting or fed states. Koren and Holmes [19] studied weight changes in patients who were receiving VNS for the treatment of medically refractory epilepsy: no significant weight changes were noted during the two years after VNS implantation and no relationship was found between changes in seizure frequency and the effect on body weight. In our study no patients suffered from this side effect.

There was also an improvement in QOL for all patients, except in two cases. These improvements involved each aspect of QOL considered (alertness, verbal communication, memory, school/professional achievement, mood, post-ictal state, seizure clustering) and were independent from the efficacy of VNS [6,21]. In addition, further observations of QOL were suggested by the decreased seizure severity, the fact that seizures were mostly nocturnal with fewer or no drop attacks, the reduction in the utilization of healthcare services, and the time spent on epilepsy-related tasks. In conclusion, despite the small group of patients studied, our experience lends a small but clear support to confirming the efficacy and safety of VNS in drug resistant partial and generalized epilepsy at developing age, but we need a larger group of patients to report more details on the response of individual seizure types.

## Competing interests

The authors declare that they have no competing interests.

## Authors' contributions

EF conceived of the study and coordination, VG participated in the design of the study and in the follow up of the patients, MCC participated in the design of the study, DB participated in the design of the study and in the follow up of the patients, IC and LI conceived the design of the figures, FCM and VM revised the language and the reference organization. DMC participated in the design of the study. All authors have read and approved the final manuscript.
